# Mild intrauterine hypoperfusion reproduces neurodevelopmental disorders observed in prematurity

**DOI:** 10.1038/srep39377

**Published:** 2016-12-20

**Authors:** Makiko Ohshima, Jacques-Olivier Coq, Kentaro Otani, Yorito Hattori, Yuko Ogawa, Yoshiaki Sato, Mariko Harada-Shiba, Masafumi Ihara, Masahiro Tsuji

**Affiliations:** 1 Department of Regenerative Medicine and Tissue Engineering, National Cerebral and Cardiovascular Center, Osaka 565-8565, Japan; 2 Institut de Neurosciences de la Timone, UMR7289, CNRS, Aix Marseille Université, Marseille 13005, France; 3 Department of Stroke and Cerebrovascular Diseases, National Cerebral and Cardiovascular Center, Osaka 565-8565, Japan; 4 Division of Neonatology, Center for Maternal-Neonatal Care, Nagoya University Hospital, Nagoya 466-8550, Japan

## Abstract

Severe intrauterine ischemia is detrimental to the developing brain. The impact of mild intrauterine hypoperfusion on neurological development, however, is still unclear. We induced mild intrauterine hypoperfusion in rats on embryonic day 17 via arterial stenosis with metal microcoils wrapped around the uterine and ovarian arteries. All pups were born with significantly decreased birth weights. Decreased gray and white matter areas were observed without obvious tissue damage. Pups presented delayed newborn reflexes, muscle weakness, and altered spontaneous activity. The levels of proteins indicative of inflammation and stress in the vasculature, i.e., RANTES, vWF, VEGF, and adiponectin, were upregulated in the placenta. The levels of mRNA for proteins associated with axon and astrocyte development were downregulated in fetal brains. The present study demonstrates that even mild intrauterine hypoperfusion can alter neurological development, which mimics the clinical signs and symptoms of children with neurodevelopmental disorders born prematurely or with intrauterine growth restriction.

In the past three decades, the incidence of mild brain injuries associated with prematurity, i.e., preterm birth (born at <37 weeks of gestation) and low birth weight (LBW, <2500 g), and intrauterine growth restriction (IUGR, birth weight <10^th^ percentile for gestational age) has been increasing^1^,^2^,^3^. Mild brain injuries include decreased white matter volume and cortical thickness with no obvious periventricular leukomalacia (PVL) or focal lesions. Infants with mild brain injuries frequently present with mild neurodevelopmental disorders (NDDs), such as attention-deficit/hyperactivity disorder (ADHD) and borderline intellectual functioning^4^. Mild NDDs associated with prematurity and IUGR have been increasing, and this phenomenon is now recognized as a critical social issue in developed countries; approximately 20% of very LBW (<1500 g) infants and 50% of extremely LBW (<1000 g) infants exhibit mild NDDs^5^,^6^,^7^. In contrast, the incidence of severe brain injuries associated with prematurity and IUGR, such as cystic PVL, has been decreasing because of improved medical care^1^,^2^,^3^. Similarly, the incidence of severe neurological sequelae associated with prematurity and IUGR, such as cerebral palsy, have also been decreasing^8^.

The etiology of preterm birth, LBW, and IUGR is multifactorial, and the identifiable causes of these three conditions largely overlap. Placental hypoperfusion and intrauterine infection/inflammation are considered the two major causes^9^. In many cases in clinical practice, the etiology is difficult to identify and may be a combination of several causes. Thus, the impact of intrauterine hypoperfusion alone, especially mild hypoperfusion, on brain development is still unclear.

The influence of severe intrauterine ischemia on the immature brain has been thoroughly explored, as most animal studies on intrauterine ischemia have been performed using models that involve the complete blockade of the blood supply to the uterus or fetus. In contrast, the influence of mild intrauterine hypoperfusion (MIUH) on the immature brain has barely been explored as there is no established model for MIUH. We previously used a model involving a suture ligation of an ovarian artery^10^. To better replicate clinically relevant prenatal hypoperfusion, in the present study, we mildly reduced blood flow to the uteri of pregnant rats via stenosis of the ovarian and uterine arteries using metal coils. Of all types of birth, extremely premature births (<28 weeks of gestation) have the highest risk for neurological sequelae; therefore, we tried to replicate the conditions of MIUH prior to 28 weeks of gestation. We subjected pregnant rats to MIUH on embryonic day 17 (E17) because E17 in rats is considered to be equivalent to embryonic weeks 20–25 in humans^11^. This novel model allows us to accurately assess the effects of MIUH on neurological development and the underlying mechanisms that cause NDDs. The aim of the present study was to evaluate the impact of MIUH on physical, neurological and behavioral development using our novel rodent model of MIUH.

## Results

### Intrauterine blood flow measurements

**Figure 1:**
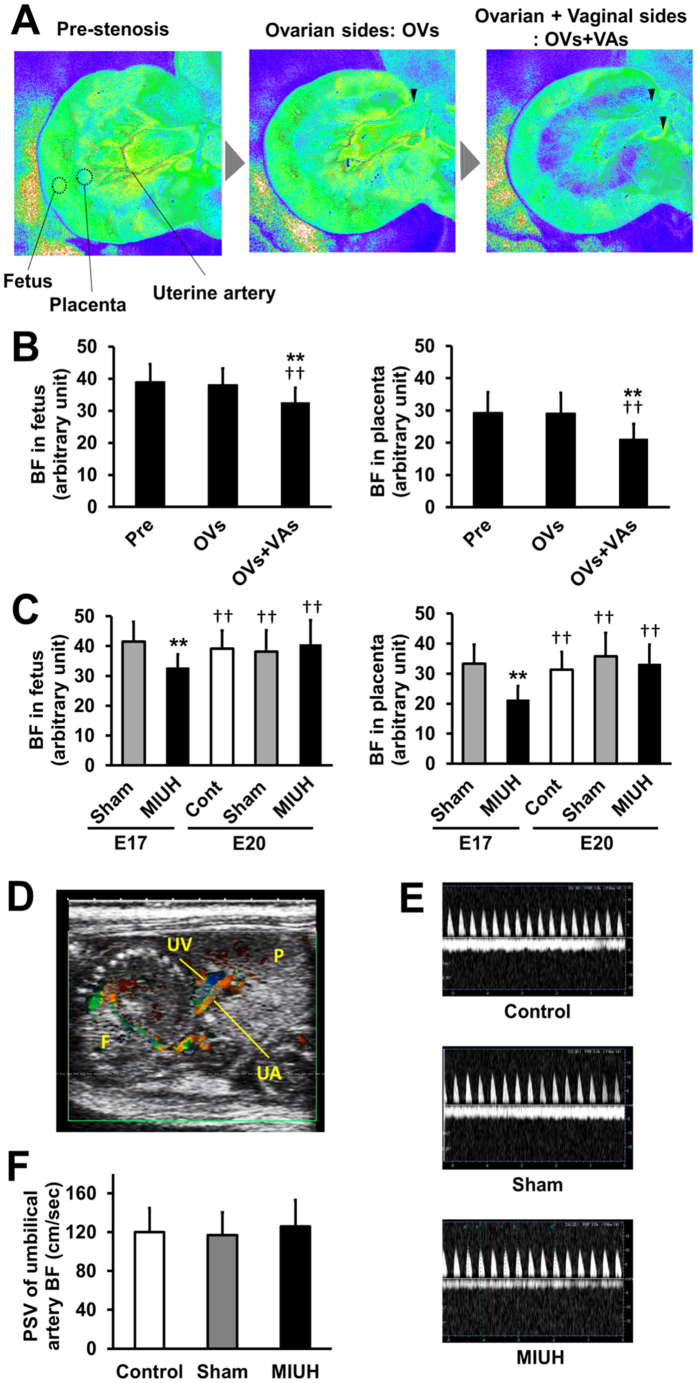
Decrease in uterine blood flow after coil stenosis in the arteries. (**A**) Representative image of changes in uterine blood flow during arterial stenosis from pre-stenosis to stenosis on the ovarian side only (OVs) and then to stenosis on both the ovarian and vaginal sides (OVs + VAs) (i.e., both the ovarian and uterine arteries). Mild intrauterine hypoperfusion (MIUH) was induced via coil stenosis at the OVs+VAs. For quantitative measurements, the regions of interest for each mouse were the fetuses and placenta. (**B**) Mean blood flow changes during the surgery in the fetuses and placentas. The blood flow in the fetuses and placentas decreased significantly immediately after coil stenosis on both the ovarian and vaginal sides compared with flow during pre-stenosis and stenosis only on the ovarian side. **P < 0.01, vs. Pre-stenosis. ^††^*P *< 0.01, vs. OVs. (**C**) Mean blood flow changes in each group immediately after surgery (E17) and three days after surgery (E20) in the fetuses and placentas. (*n* = 3 dams, 22-47 fetuses, and 18-47 placentas in each group at each time point). **P < 0.01, vs sham at E17. ^††^*P* < 0.01, vs. MIUH at E17. Pre: pre-stenosis. Cont: control. (**D**) Representative ultrasound imaging of blood flow in the umbilical cord artery (UA) and vein (UV). P indicates placenta, and F indicates fetus. (**E**) Comparison of the peak systolic velocity (PSV) of blood in the umbilical cord artery determined via pulsed-wave Doppler imaging. Y-axis, umbilical cord blood velocity (cm/s). (**F**) Summary of the mean PSV among groups (*n* = 12 in each groups). The PSVs were not different among groups at E19. PSV: peak systolic velocities. BF: blood flow.

Pregnant Sprague-Dawley rats were subjected to MIUH on E17. We previously developed a technique to reduce carotid artery blood flow using metal microcoils^12^. We applied these microcoils to all of the feeding arteries to the uterus, i.e., the bilateral uterine and ovarian arteries, so that all fetuses were subjected to comparable levels of hypoperfusion. Microcoil stenosis only in the ovarian artery did not decrease blood flow to the fetuses and placentas compared with the pre-stenosis level (Fig. 1A). Microcoil stenosis in both the ovarian and the uterine arteries significantly decreased blood flow to the fetuses and placentas to 84 and 72%, respectively, of the pre-stenosis level (Fig. 1B). The blood supply after the application of four-coil stenosis was mostly the same across each fetus (arbitrary units of blood flow measurement: mean ± SD: 32.7 ± 4.5) and each placenta (21.2 ± 4.6). Uterine blood flow was restored to pre-stenosis levels 3 days after the surgery, on E20 (Fig. 1C). Color Doppler ultrasonography demonstrated that the peak systolic blood velocities in umbilical arteries 2 days after the surgery, i.e., E19, were almost the same among the three groups (Fig. 1D–F). Mean blood velocities in umbilical veins were also almost identical among the groups. These findings suggest that the blood supply from the placenta to fetuses was not impaired by MIUH 2 days after the surgery. Our new technique, microcoil stenosis in all four feeding arteries to the uterus on E17, produced MIUH that was consistent across all placentas and fetuses. The extent of hypoperfusion was significantly less severe in the fetuses than in the placentas (*P* < 0.001) and was resolved within 3 days.

### Physical development

**Figure 2:**
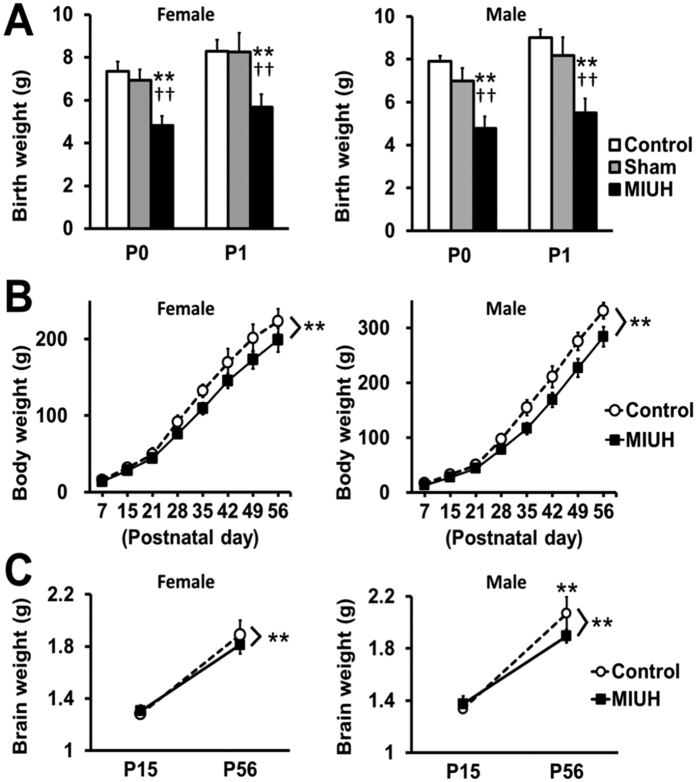
Body weight and brain weight. (**A,B**) The body weight was repeatedly measured from birth to adulthood. In both female and male pups, pups exposed to mild intrauterine hypoperfusion at E17 (MIUH group) were born with remarkably low birth weights. In addition, the rats in the MIUH group had significantly reduced body weights compared with the weights of the control rats throughout the observation period, i.e., up to P56 (control group, females *n* = 8 and males *n* = 10; sham group, females *n* = 9 and males *n* = 14; MIUH group, females *n* = 9 and males *n* = 12). (**C**) Brain weights were measured P15 and P56. Two-way repeated-measures ANOVA revealed that the mean brain weights of female and male rats in the MIUH group were significantly lower than those of the controls. Post-hoc analysis showed that the brain weight of males in the MIUH group was markedly decreased compared with that of the control group at P56. (*n* = 8–16 in each group, each sex and each time point) **P < 0.01 vs. control group. ^††^*P* < 0.01 vs. sham group. E, Embryonic day. P, Postnatal day.

Compared with the sham and control groups, the pups with MIUH exhibited significantly shorter gestational periods; pups in the control group were born on E23, whereas those in the sham group were born 1 day earlier (E22), and those in the MIUH group were born 1–2 days earlier (E21-22) via spontaneous labor (Table 1). The mortality rate in the sham group was 8% at the endpoint; 23 of 25 fetuses identified during the laparotomy were born and survived until the end of the observation period. The mortality rate in the MIUH group was 17% at the endpoint; 43 of 47 fetuses identified during the laparotomy were born, and 39 survived until the end of the observation period. Compared with the control and sham groups, the MIUH group showed significantly decreased birth weights for both female and male pups (Fig. 2A). Intrauterine blood flow, blood flow velocity in the umbilical artery, and the birth weights of the female and male pups were not different between the control and sham groups; therefore, in subsequent experiments, we compared the MIUH group with the control group. The low body weight of rats in the MIUH group persisted until adulthood (Fig. 2B). Two-way repeated-measures ANOVA revealed that the mean brain weights of the female and male rats in the MIUH group were significantly lower than those of the control group (Fig. 2C). These results demonstrate that MIUH causes IUGR, premature birth, LBW, and growth retardation after birth.

### Behavioral tests

**Figure 3:**
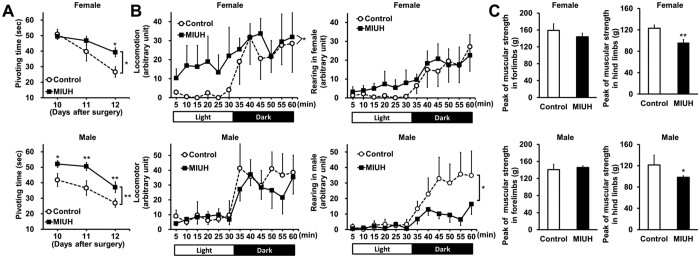
Behavioral tests. (**A**) To examine neonatal reflexes, negative geotaxis tests were performed on three consecutive days from 10 to 12 days after the surgery (control group, female *n* = 8 and male *n* = 10; MIUH group, female *n* = 17 and male *n* = 22). The development of neonatal reflexes in female and male pups exposed to mild intrauterine hypoperfusion on E17 (MIUH group) was significantly slower than that of the control pups. (**B**) To measure spontaneous activity, P15 rats were subjected to an open-field test. For locomotor activities (horizontal movements), female rats exposed to MIUH showed significant hyperactivity compared with the activity of control rats in a light environment. For rearing activities (vertical movements), male rats in the MIUH group showed hypoactivity compared with the activity of control rats in a dark environment (control group, female *n* = 8 and male *n* = 6; MIUH group, female *n* = 9 and male *n* = 12). (**C**) Muscular strength in the forelimbs and hind limbs was measured using a traction meter on P35. For the hind limbs, muscular strength was significantly lower in the female and male rats exposed to MIUH than in the control rats. For the forelimbs, no clear difference between the two groups was detected (control group, female *n* = 5 and male *n* = 3; MIUH group, female *n* = 9 and male *n* = 12). ^*^P < 0.05, ^**^P < 0.01, vs. control group. E, Embryonic day. P, Postnatal day.

To investigate neonatal physiological reflexes, a negative geotaxis test was performed on three consecutive days. Pivoting times became shorter each day in the control group. Two-way repeated-measures ANOVA revealed that the pivoting time was significantly longer for the MIUH group than for the control group in both female and male pups (Fig. 3A). To measure spontaneous activity during childhood, the rats were evaluated in an open-field test on postnatal day 15 (P15). Compared with control rats, female rats exposed to MIUH showed significant locomotor hyperactivity in a light environment; in contrast, male rats showed reduced rearing activity in a dark environment (Fig. 3B). Compared with the control group, both female and male juvenile rats in the MIUH group showed significant muscle weakness in their hind limbs but not in their forelimbs (Fig. 3C). These results demonstrate that this MIUH model produces alterations in several aspects of neurological development.

### Neuroanatomical changes

**Figure 4:**
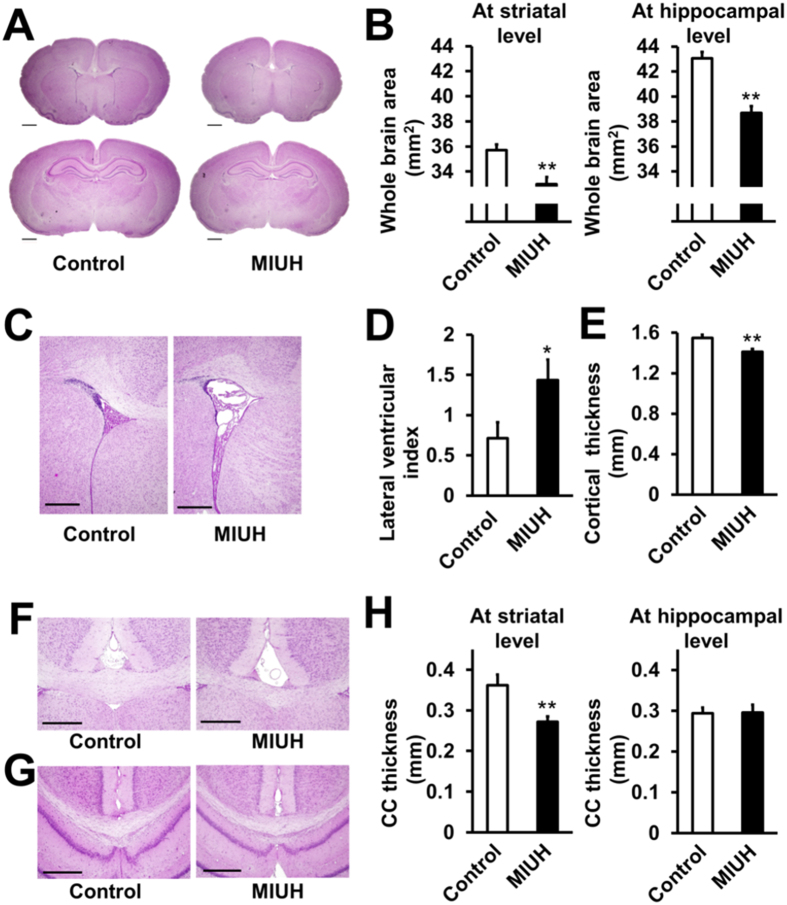
Histological analysis with H&E stain. (**A–H**) Hematoxylin & eosin (H&E)**-**stained sections at P15 were used for neuroanatomical analyses. (**A**) Representative images of coronal sections at the striatal and hippocampal levels. The scale bar represents 1 mm. (**B**) The hemispheric areas at both the striatal and hippocampal levels were significantly reduced in the MIUH group compared with the areas in the control group. (**C**) Representative images of the lateral ventricles at the striatal level. The scale bar represents 500 μm. (**D**) The lateral ventricular index was calculated as the percent of the lateral ventricular area relative to hemispheric area at the striatal level. The lateral ventricles were significantly enlarged in the MIUH group compared with those in the control group. (**E**) Cortical thickness at the striatal level. The cortical thickness was decreased in the MIUH group. (**F**) Representative images of the corpus callosum at the striatal level. (**G**) Representative images of the corpus callosum at the hippocampal level. The scale bar represents 500 μm. (**H**) The median thickness of the corpus callosum was significantly reduced at the striatal level in the MIUH group. *n* = 17 in the control group, and *n *= 18 in the MIUH group. ^*^P < 0.05, ^**^P < 0.01 vs. the control group.

**Figure 5:**
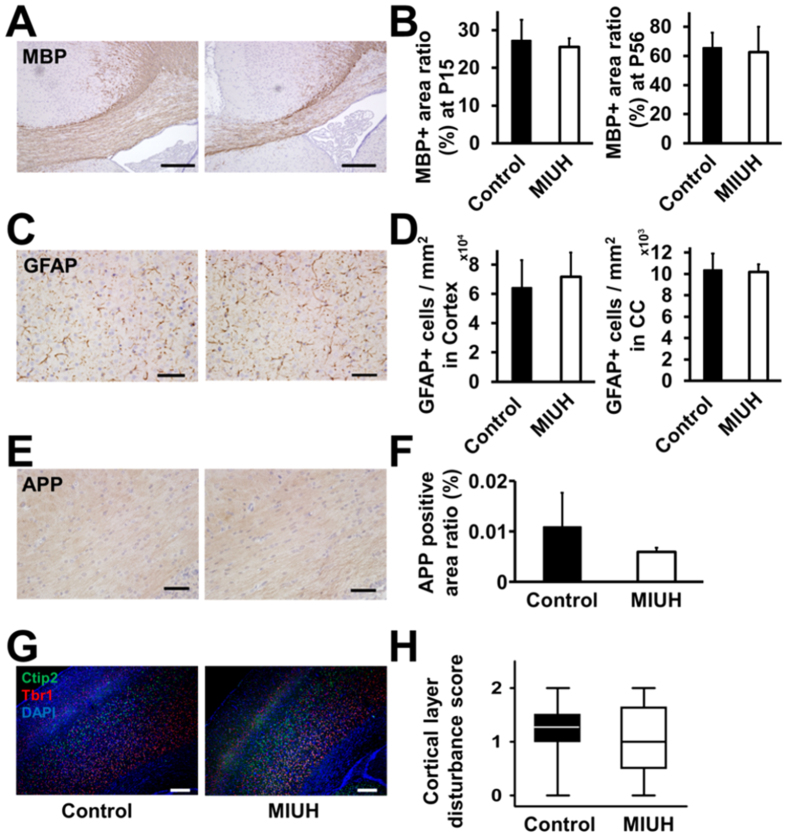
Histological analysis with immunostaining. (**A**) Representative images of immunohistochemical staining with the oligodendrocyte maker MBP in P15 rat brains. (**B**) The ratios of areas with MBP-positive staining in the corpus callosum at P15 and P56 (control group, *n* = 6 at P15 and *n* = 16 at P56; MIUH group, *n* = 5 at P15 and *n* = 22 at P56). (**C**) Representative images of immunohistochemical staining with the astrocyte maker GFAP at P15. (**D**) The numbers of GFAP-stained cells in the cortex and corpus callosum (control group, *n* = 6; MIUH group, *n* = 6). (**E**) Representative images of immunohistochemical staining with the axonal injury marker APP at P15. (**F**) The ratios of areas with APP-positive staining in the corpus callosum at P15 (control group, n = 6; MIUH group, n = 6). No significant differences were found between the control and MIUH groups with respect to the ratios of positively stained area or the number of stained cells. The scale bars represent 250 μm in A and 50 μm in (**C** and **E**). (**G**) Representative images of the cortical layer at P15. The VI layer was stained with anti-TBR1 (red) antibody, the V layer was stain with anti-Ctip2 (green) antibody, and the nuclei were stained with DAPI (blue). (**H**) Disturbances in the cortical layer were not detected in the rat brains exposed to MIUH on embryonic day 17 based on semi-quantitative scoring (median ± quartiles) (control group, *n* = 16; MIUH group, *n* = 18). The scale bar represents 250 μm. P, Postnatal day. MIUH, mild intrauterine hypoperfusion.

At P15, the cerebral area in the MIUH group was significantly smaller than that in the control group both at the striatal and hippocampal levels (Fig. 4A,B). The lateral ventricular index in the MIUH group was significantly higher than that in the control group (Fig. 4C,D). The thicknesses of the cortex and the corpus callosum at the striatal level were significantly decreased compared with those in the control group (Fig. 4E–H). H&E staining did not show obvious tissue damage, such as cystic and necrotic lesions, cell loss or inflammatory cell infiltration. The ratios of the MBP-, GFAP- and APP-positive areas in the corpus callosum and the cortex were not significantly different between the MIUH and control groups (Fig. 5A–F). Semi-quantitative scores of the level of disturbance in cortical structures did not differ between the groups (Fig. 5G,H). Similar, but less extensive alterations were observed at P56, i.e., adulthood. The cerebral area in the MIUH group (70.6 ± 2.3 mm^2^) was significantly smaller than that in the control group (74.8 ± 6.3 mm^2^) at the hippocampal level but not at the striatal level. The thickness of the corpus callosum at the striatal level (0.38 ± 0.07 mm) was decreased but was not significantly different from that in the control group (0.44 ± 0.13 mm, *P* = 0.08). There were no significant differences between the two groups with respect to cortical thickness, the ventricular index, or the ratio of the MBP-positive areas in the corpus callosum (Fig. 4B) at P56. Taken together, these data indicate that MIUH results in impaired morphological brain development, i.e., decreased cerebral hemispheric area, decreased gray and white matter areas, and enlarged lateral ventricles, all of which are typically observed in the brains of prematurely born infants. However, MIUH did not cause clear tissue or cellular damage in mature myelin, astrocytes, and axons and did not disturb the gross structure of cortical layers.

### Changes in protein levels in the sera of dams, amniotic fluid, placentas, and fetal brains

**Figure 6:**
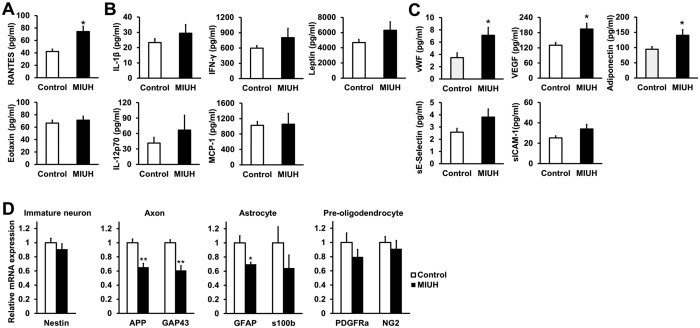
Levels of inflammatory cytokines and chemokines and ischemia-related proteins in the placenta on E20. The levels of several inflammatory and ischemia-related factors were measured via a multiplex assay. (**A**) Inflammatory chemokines. RANTES levels were significantly increased, whereas eotaxin levels did not change in the placenta 3 days after coil stenosis. (**B**) Inflammatory cytokines. Cytokine levels did not significantly change in the placenta following mild intrauterine hypoperfusion (MIUH) on E17, although all inflammatory cytokines showed increasing trends. *n* = 11 in the control group and *n* = 12 in the MIUH group. (**C**) Ischemia-related proteins. The levels of vWF, VEGF, and adiponectin were significantly increased in the placenta following MIUH. *n* = 13 in the control group and *n* = 14 in the MIUH group. ^*^P < 0.05, vs. the control group. (**D**) mRNA expression levels of an immature neuron maker (nestin), axonal makers (APP and GAP43), astrocyte makers (GFAP and S100β), and oligodendrocyte makers (PDGFRα and NG2) in the whole brain (*n* = 5 in each group). β-actin was used as an internal control for gene expression. The mRNA expression levels of APP, GAP-43 and GFAP in brains on E20 were significantly decreased following MIUH. ^*^P < 0.05, ^**^P < 0.01 vs. the control group. E, Embryonic day.

To elucidate the mechanisms underlying prenatal brain injury and to identify the biomarkers of MIUH-induced brain injury, changes in protein levels in the sera of dams, amniotic fluid, placentas and fetal brains were investigated using a multiplex protein assay on E20. In the sera, amniotic fluid and brains, there was no clear changes in the MIUH group compared with the control group for all chemokines, cytokines and ischemia-related proteins. In contrast, most chemokine levels in the placentas showed an increasing trend in the MIUH group. Among inflammation-related chemokines and cytokines, RANTES levels were significantly increased in the MIUH group (Fig. 6A,B). Among ischemia-related proteins, vWF, VEGF, and adiponectin levels were significantly elevated in the MIUH group (Fig. 6C). Thus, MIUH has a deleterious impact, mainly in the placenta, on the levels of proteins related to inflammation and ischemic/vascular injury.

### mRNA expression in the fetal brain

To investigate the damage that MIUH may cause to immature neural cells, the mRNA expression of nestin (an immature neuron marker), APP and GAP-43 (axon makers), PDGFRα and NG2 (pre-oligodendrocyte markers), and S100β and GFAP (astrocyte markers) was measured in fetal brains on E20. Expression in the MIUH group decreased by 39% for GAP43, 34% for APP, and 30% for GFAP compared with expression in the control group. There were no differences in the mRNA expression of nestin, PDGFRα, NG2, or S100β between the two groups (Fig. 6D). These results indicate that MIUH on E17 may cause incomplete axonal outgrowth and astrocytes dysfunction but no or little impairment of immature neurons and pre-oligodendrocytes. These observations clearly coincide with the maturation patterns of neural-lineage cells in the fetal brain around E17, when the generation of new neurons is in its last stage, the generation of new astrocytes is in its early stage, and the generation of new oligodendrocytes has not commenced^13^.

## Discussion

The present study focuses on how MIUH affects neurological development in a novel rodent model. MIUH controlled by microcoil stenosis in rats successfully reproduced the IUGR, premature birth, cerebral maldevelopment, and behavioral alterations observed in preterm children. In particular, we found that MIUH induced (1) low body weight; (2) a delayed neonatal reflex, reduced muscle strength and altered spontaneous activity; (3) macroscopic morphological changes, including ventricular enlargement and the thinning of the cortex and corpus callosum in the childhood stage, and reduced brain weight; and (4) early changes marked by the upregulation of several proteins related to inflammation and ischemic injury in the placenta and the downregulation of mRNAs associated with axon and astrocyte growth, but not in those associated with neuron or oligodendrocyte growth, in the fetal brains.

To our knowledge, there is no rodent model of MIUH apart from the model based on suture ligation of the uterine artery that we used previously^10^,^14^,^15^,^16^,^17^,^18^. In contrast, there are many models of total intrauterine ischemia that have been developed to study prenatal brain injury^19^,^20^,^21^,^22^,^23^. As a result, brain injuries caused by the total reduction of intrauterine perfusion has been well delineated, whereas very little is known regarding the impact of MIUH. Although rodent models of suture ligation have been reported as appropriate models to investigate the influence of hypoperfusion, these models have some limitations: the degree of hypoperfusion is unknown, there is huge inter-fetal variability and the possibility of fetal death, and there may be a selection bias because only pups with severe LBW are chosen for evaluations. In this study, metal microcoils were wrapped around the feeding arteries to the uterus to induce fetal hypoperfusion. Using such microcoils allowed us to control and optimize the extent to which blood flow was reduced, producing minimal intra-animal variation and low mortality and enabling accurate evaluations. The blood flow reduction in both the placenta and the fetus was mild (approximately 30% of the pre-stenosis level in the placenta) and was restored to a normal level within 3 days after the surgery. Nevertheless, our study clearly demonstrates that MIUH induces long-lasting changes in neurological development. The limitations of this model are the long anesthesia time and the need for laparotomy, both of which are not experienced by pregnant women or human fetuses.

The MIUH model resulted in several changes in neuroanatomy and behavior, many of which are similar to those observed in children born preterm or with IUGR. (1) All the pups experiencing MIUH weighed more than 2 SDs below the mean control value at birth. (2) The low body weight in the animals with MIUH did not normalize until reaching young adulthood, which is commonly observed in patients with IUGR^24^. (3) MIUH resulted in lower brain weights and decreased cortex and white matter areas, which are common features observed via magnetic resonance imaging (MRI) in patients with IUGR^25^,^26^. (4) Lateral ventricular enlargement was also observed in the present model and is also frequently found in patients with IUGR and VLBW^27^. (5) MBP, GFAP and APP expression measured via immunohistochemistry at either childhood or adulthood did not change as strongly as has been shown in other models of severe intrauterine ischemia or PVL models^10^,^28^. The anatomical findings outlined above in items 3–5 in this model are similar to those seen in patients with diffuse white matter injury, which is currently the most frequently observed pattern of brain injury in premature infants. Diffuse white matter injury takes the form of either non-cystic PVL or cerebral white matter abnormalities^29^, and the latter form is characterized by diffuse astrogliosis without focal necrosis^30^. Hence, this MIUH model represents the mild end of the spectrum of brain injuries observed in association with prematurity, which extends from severe cystic PVL to minimal injuries. (6) MIUH also delayed the development of neonatal physiological reflexes, illustrated by negative geotaxis. The delayed development of neonatal reflexes is known to be associated with dysfunctions of the central nervous system in human infants^31^. (7) MIUH induced hyperactivity in a light environment in females, suggesting similarities with the ADHD-like symptoms observed in children. In children with ADHD, a thinned cortex has been reported via MRI^32^, and our MIUH model also resulted in decreases in cortical thickness. (8) MIUH also caused moderate muscle weakness in the hind limbs. The findings outlined above in items 6–8 demonstrate that this MIUH model causes mild but significant behavioral abnormalities, suggesting that MIUH may be used as a model of mild NDDs as opposed to models of severe brain damage, such as cerebral palsy. Taken together, these results show that MIUH disturbs physical, neuroanatomical and behavioral development. Notably, MIUH did not induce obvious disruptions in brain histology despite obvious alterations in behavior and gross brain morphology. As the degree of low birth weight was more conspicuous than the reductions in gestational periods, this model is more reflective of IUGR than preterm birth. More details on the phenotype caused by this model, however, are required to establish this as an IUGR model.

Low blood flow in the feeding arteries to the uterus leads to placental dysfunction and subsequently induces fetal growth restriction^33^,^34^; however, few studies have explored the molecular mechanisms leading to developmental disorders or have evaluated placental and fetal conditions separately. In the present study, we investigated the molecular changes induced by MIUH in four distinct tissues, i.e., maternal blood serum, placenta, amniotic fluid and fetal brain.

Hypoperfused placentas showed consistently higher levels of inflammatory chemokines than control placentas, whereas cell adhesion molecules known to be upregulated by inflammatory cytokines^35^, i.e., sICAM-1 and sE-selectin, showed no changes. The inflammatory cytokine levels might have been insufficient to upregulate these cell adhesion molecules. MIUH upregulated vWF, VEGF and adiponectin levels, which are known endothelial injury markers and have been reported to be candidate biomarkers for preeclampsia^36^,^37^. Adiponectin suppresses insulin signaling and the uptake of insulin-stimulated amino acids in the placenta, and its regulation has important implications for nutrient transport in the placenta^38^,^39^,^40^. Therefore, increased adiponectin levels may disturb nutrient transport to the fetus across the placenta. The present study demonstrates that placental function deteriorates in response to MIUH. The altered protein levels in the placenta correspond to clinical findings in pregnant women with placental dysfunction, which leads to prematurity, IUGR and subsequently to NDDs. Notably, these protein levels were not altered in the sera or amniotic fluid of dams, suggesting that those samples may not be appropriate for detecting biomarkers of mild fetal injury in clinical practice and that MIUH mainly affects the placenta. The influence of the surgical procedure cannot be ruled out as the protein levels were not measured in the sham group.

Neurogenesis and gliogenesis are essential for normal brain development. Thus, we investigated the expression of several mRNAs related to brain development in the late gestational period, E20, when uterine blood flow had returned to normal. MIUH significantly decreased the mRNA levels for proteins essential for the migration of cortical neurons (APP), axonal outgrowth (GAP-43), and astrocyte maturation (GFAP)^41^,^42^,^43^,^44^. Axonal outgrowth in the developing brain originates in the cortical plate and begins at approximately E13 in rats^45^. The decrease in axonal outgrowth-related mRNA (GAP-43) expression in the present study indicates that MIUH interferes with the typical development and refinement of the brain on E17. Although astrocytogenesis mainly occurs after birth in rats, it is initiated on the medial walls of the lateral ventricles on E18 in rats^46^. The decreased GFAP mRNA expression along with the lateral ventricle enlargement indicates that MIUH induces alterations in the astrocytogenesis around the ventricles. Despite the decreased mRNA levels for APP, GAP-43, and GFAP at E20, immunohistological evaluations on P15 or P56 showed no marked defects in staining for APP, GFAP, MBP, and TBR1 and Ctip2 (cortical layer makers) proteins. This lack of changes in the later stage of development may be due to plasticity in immature brains from E20 to P15. However, more detailed examinations of neuronal maturation associated with the subplate or the microstructures of axons and astrocytes may reveal subtle abnormalities at P15 and P56. The reduced gray and white matter areas, at least at P15, indicate that the development of neural and glial structures was certainly altered.

In conclusion, MIUH induced placental insufficiency, leading to long-lasting alterations in brain development. These mild neuroanatomical and behavioral changes observed in our MIUH model reflect several, if not all, of the mild neurological impairments found in infants born with IUGR, LBW, and mild prenatal brain injury. Further studies are needed to better delineate the alterations MIUH may cause and the mechanisms related to MIUH that lead to neurological deficits; such as cognitive functioning at adulthood and damage to the subplate in the cortex. Our novel, promising model of MIUH may contribute to a better understanding of the early mechanisms leading to cerebral alterations associated with prematurity and the development of related NDDs, as well as to the development of new strategies for neuroprotection that focus on the placental compartment.

## Materials and Methods

### Arterial stenosis using microcoils

**Figure 7:**
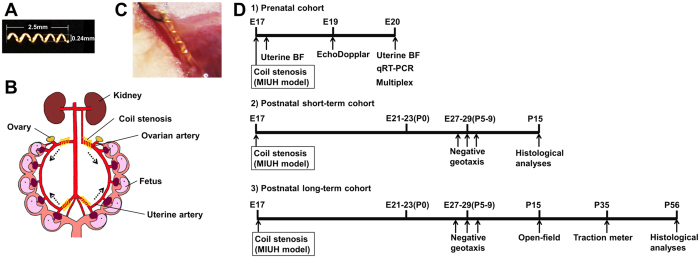
Schema of microcoil and experimental procedures. (**A**) Image of a microcoil. Coils were 2.5 mm long with five turns, and the inner diameter was 0.24 mm. (**B**) Schema of coil stenosis. Four microcoils were installed around the arteries upstream of the bilateral uterine and ovarian arteries. The dotted arrows indicate the direction of arterial blood flow. (**C**) Pictures of coil stenosis in a pregnant SD rat on E17. The decreased blood flow inside the coil was observed under a microscope. (**D**) Experimental design. Three experimental animal cohorts were used. (1) Prenatal cohort, (2) Postnatal short-term cohort, (3) Postnatal long-term cohort. E, Embryonic day. P, Postnatal day. MIUH, mild intrauterine hypoperfusion. qRT-PCR, quantitative real-time PCR. BF, blood flow.

All experiments were performed in accordance with protocols approved by the Experimental Animal Care and Use Committee of the National Cerebral and Cardiovascular Center, Suita, Japan. Twenty pregnant Sprague-Dawley rats (Japan SLC Inc., Shizuoka, Japan) were prepared. MIUH was induced via arterial stenosis on E17 under isoflurane anesthesia. We used microcoils (φ = 0.24 mm) made from gold-coated steel (SAMINI Co. Ltd., Shizuoka, Japan, Fig. 7A). The inner diameter of the microcoils was optimized to induce LBW but not abortion in response to a severe reduction in fetal blood flow. Four feeding arteries to the uterus, i.e., the bilateral uterine and ovarian arteries (Fig. 7B), were exposed and separated from the veins running along each artery to provide space for microcoil insertion. Each artery was gently lifted with a silk suture, and the microcoil was wrapped around the artery by rotating 5 times under a microscope. Blood flow reduction inside the artery was confirmed visually based on the whitening of the artery (Fig. 7C). The sham group was subjected to the same surgery as the MIUH group but without microcoil placement (sham group), and dams that did not undergo surgery were prepared as a control group.

### Experimental cohorts

We established three experimental cohorts of animals. For the Prenatal cohort, three dams in each group underwent blood flow measurement via color Doppler ultrasonography and laser speckle flowmetry. Next, the dams were euthanized on E20 for mRNA and protein expression analyses. In the two Postnatal cohorts, pups were delivered via spontaneous labor. The day of birth was considered postnatal day 0 (P0). Pups underwent serial body weight measurements and behavioral tests until the experimental endpoint. The pups were euthanized for histological analyses at P15 (Postnatal short-term cohort) or P56 (Postnatal long-term cohort) (Fig. 7D). The numbers of individuals in each group were as follows: Prenatal cohort, MIUH group, *n* = 3; sham group, *n* = 3; and control group, *n* = 3: Postnatal cohorts, MIUH group, *n* = 4 (female pups *n* = 17, male pups *n* = 23); sham group, *n* = 2 (female pups *n* = 9, male pups *n* = 14); and control group, *n* = 5 (female pups *n* = 24, male pups *n* = 24). Littermates were randomly divided into two Postnatal cohorts: Postnatal short-term and Postnatal long-term cohorts.

### Laser speckle flowmetry

Temporal changes in intrauterine blood flow during surgery were monitored using laser speckle flowmetry (Omegazone, Omegawave Inc, Tokyo, Japan) at three time points, i.e., before stenosis, after stenosis on the ovarian artery only, and after stenosis on both the ovarian and uterine arteries (Fig. 1A). Intrauterine blood flow was measured again on E20. For quantitative analyses, regions of interest (ROIs) in all fetuses or placentas (left image in Fig. 1A) were analyzed.

### Color-Doppler ultrasonography

Blood flow velocities in the umbilical artery and vein on E19 were determined via pulsed-wave Doppler imaging. Pregnant rats were anesthetized with isoflurane and were placed in a supine position. Blood flow was visualized using an Aplio 80 equipped with a 14-MHz PLT-1202S transducer (Toshiba Medical Systems Corp, Tochigi, Japan, Fig. 2D). Umbilical cords that had a Doppler angle of less than 60 degrees were selected for blood flow measurements. The overall gain setting was optimized at the beginning of each recording session. The peak systolic velocity and mean velocity for blood flow in the umbilical artery were measured.

### Negative geotaxis test

To evaluate sensorimotor and reflex development, negative geotaxis tests were performed for three consecutive days from 10 to 12 days after the surgery, i.e., P4 to P6 if the pups were born on E23. To avoid an influence of different gestational periods on each litter, the timing of the tests was determined based the time since surgery rather than since birth. The animal was placed on a 25-degree incline board with its head facing downwards. Negative geotaxis was defined as the time in seconds required for the animal to turn 180° and begin crawling up the slope, and each animals was observed for up to 60 seconds.

### Open-field test

Open-field tests were performed on P15, as previously described^47^. Briefly, animals were allowed to freely explore a box (30 × 30 cm) for 30 min in a light environment and for a subsequent 30 min in a dark environment. Infrared beams were mounted at specific intervals along the X, Y, and Z axes of the box. The total number of beam crossings by the animal was counted and scored as “locomotion” for horizontal movements and as “rearing” for vertical movements.

### Traction meter

Muscle strength in the forelimbs and hind limbs was assessed on P35 using a traction meter with steel grids (Brain Science Idea, Co., Ltd, Osaka, Japan). Each rat was placed on the steel grid of the apparatus, and an experimenter slowly pulled the tail backwards parallel to the surface of the grid at a constant speed. Five successful strength measurements in both the forelimbs and the hind limbs were recorded, and the average peak strength was analyzed using BS-TM-SOF software (Brain Science Idea, Co., Ltd).

### Real-time quantitative PCR

Total RNA was extracted from isolated fetal brains on E20 using an RNeasy Mini kit (https://www.qiagen.com/us/, Qiagen, Hilden, Germany). The obtained RNA was reverse-transcribed into cDNA using a Quantitect Reverse Transcription kit (Qiagen). PCR amplification was performed on a 7500 Real-Time PCR System (Applied Biosystems, Carlsbad, CA, USA) using Power SYBR Green PCR Master Mix (Applied Biosystems). β-actin was used as an internal control. The levels of β-actin in each group were confirmed to be almost the same, and equal amounts of mRNA were transcribed into cDNA for each sample. The primers are listed in Table 2 (Invitrogen, Carlsbad, CA, USA).

### Multiplex assay

The total protein in each brain and placenta was extracted with RIPA buffer (Nacalai Tesque, Kyoto, Japan) followed by homogenization, and protein concentrations were measured using a Pierce™ BCA Protein Assay Kit (Thermo Fisher Scientific Inc. MA USA). Whole blood was sampled from dams via their tail vein, incubated on ice, and centrifuged to purify the serum. Amniotic fluid was sampled from the uterus using a 25 G needle. All samples were stored at −80 °C until used in a Multiplex assay, and 25 μl of total protein (300–800 μg/ml) was used for the assay. A multiplexed immunoassay based on Luminex xMAP technology was performed using Rat Cytokine/Chemokine Magnetic Bead Panels for measuring RANTES, eotaxin, IL-1β, IL-12p70, IFN-γ, MCP-1, leptin, and VEGF (#RECYTMAG-65K https://www.emdmillipore.com/US/en, Millipore, Darmstadt, Germany) and Rat Vascular Injury Magnetic Bead Panels for measuring vWF, sE-selectin, sICAM-1, and adiponectin (#RV2MAG-26K, Millipore). Assays were performed according to the manufacturer’s instructions. Absolute values for each sample were adjusted relative to the protein concentration.

### H&E staining and immunohistochemical analyses

Animals were intracardially perfused with 4% paraformaldehyde, and their brains were removed and weighed on P15 or P56. All brains were embedded in paraffin. Sections (5-μm thick) were cut at 3 mm (striatal level) and 4 mm (hippocampal level) from the frontal tip. One section at each level from each animal was used in each histological analysis. The sections were stained with hematoxylin and eosin (H&E). The whole cerebral area, median thickness of the corpus callosum at the striatal and hippocampal levels, lateral ventricular index and cortical thickness at the striatal level were measured using ImageJ software (NIH, Bethesda, MD, USA). Lateral ventricular index values were calculated as follows: total area of the bilateral ventricles/whole area at the striatal level × 100^48^. For immunohistochemical analyses, sections at both the striatal and hippocampal levels were stained with anti-MBP, anti-GFAP or β-APP antibodies after deparaffinization, antigen retrieval with citrate buffer at pH 6 (DAKO, Glostrup, Denmark), the quenching of endogenous peroxidases and protein blocking (DAKO). The sections were then stained with anti-mouse LSAB/HRP (DAKO) or anti-rabbit Envision+ System-HRP Labeled Polymer (DAKO) and subsequently visualized with 0.5% diaminobenzidine (DAB). Sections were photographed at 200× magnification for APP- and GFAP- stained sections and at 40× magnification for MBP-stained sections using a digital microscope (BIOREVO BZ-9000; KEYENCE, Osaka, Japan). The ratios of the areas positively stained with MBP (P15 and P56) and APP (P15) in the corpus callosum and the number of GFAP (P15)-positive cells in the cortex and corpus callosum were analyzed in one section from each animal using WinROOF imaging software (Mitani Co. Ltd., Fukui, Japan). To visualize the cortical layer at P15, sections from the striatal level were incubated with rabbit anti-TBR1 and anti-Ctip2 antibodies and triple-stained with AlexaFluor 546 anti-rabbit IgG antibody, AlexaFluor 488 anti-rat IgG antibody (1:1000, Invitrogen), and DAPI (Dojindo, Kumamoto, Japan). The degree of disturbance of the cortical layers was evaluated based on scores ranging from 0 to 2 points (0, normal; 1, mildly disturbed; 2, disturbed). Information regarding all primary antibodies is listed in Table 3.

### Statistics

Comparisons among the three groups (control, sham, and MIUH groups) were assessed using one-way ANOVAs, followed by a Tukey-Kramer tests. Comparisons of two groups were assessed using Student’s *t*-tests. When analyzing semi-quantitative scores, comparisons of two groups were assessed using Mann-Whitney’s *U*-tests. Temporal changes in blood flow, body weight, brain weight, and performances in the negative geotaxis and open-field tests were assessed using two-way repeated-measures ANOVAs followed by Bonferroni-Dunn tests. Females and males were analyzed separately. The comparison of gestation periods was made using a Wilcoxon rank-sum test. Differences were considered significant at *P* < 0.05. All statistical analyses were performed using Statcel3 software (OMS Ltd. Saitama, Japan). The results are expressed as the mean ± standard error of the mean (SEM), unless otherwise noted.

## Additional Information

**How to cite this article**: Ohshima, M. *et al*. Mild intrauterine hypoperfusion reproduces neurodevelopmental disorders observed in prematurity. *Sci. Rep.* **6**, 39377; doi: 10.1038/srep39377 (2016).

**Publisher's note:** Springer Nature remains neutral with regard to jurisdictional claims in published maps and institutional affiliations.

## Figures and Tables

**Table 1:** Gestational age and number of live-birth pups and dams.

Number of Pups at P0/Dams				
Gestational age	E21	E22	E23	Total
Control group			18/2	18/2
Sham group		23/2		23/2
MIUH group	9/1	34/3		43/4

E, embryonic day; MIUH, mild intrauterine hypoperfusion

The pups experiencing MIUH exhibited significantly shorter gestational periods than the sham (P < 0.05) and control groups (P < 0.01) (Wilcoxon rank-sum test).

**Table 2:** Primers for qRT-PCR.

Gene	Forward	Reverse
Nestin	5′-AGGCCTTCAAAAGAGGGGAA-3′	5′-ACCTTGGGAAGCTCTGATCC-3′
APP	5′-CAACCGTGGCATCCTTTTGG-3′	5′-GAGTGGTCAGTCCTCGGTCA-3′
GAP-43	5′-CCGAGGCTGACCAAGAACAT-3′	5′-GGTAGGAGAGGACAGGCTCA-3′
GFAP	5′-AATTGCTGGAGGGCGAAGAA-3′	5′-TTGAGGTGGCCTTCTGACAC-3′
S100β	5′-TCCACACCCAGTCCTCTCTG-3′	5′-GAGGCTCCTGGTCACCTTTT-3′
PDGFRα	5′-AAACAGAGGAACTGTGGGCGA-3′	5′-CCCCATCGCTCCTGAGAACTT-3′
NG2	5′-TACAAGTCCAGACGCCCAAC-3′	5′-GTGGTTCTCCCCGAAGAAGG-3′
β-actin	5′-GCCCTAGACTTCGAGC-3′	5′-CTTTACGGATGTCAACGT-3′

**Table 3:** Antibodies.

Protein	Host	Dilution	Incubation time	Product supplier
MBP	Rabbit	1:400	1 h	ABBIOTEC, San Diego, CA, USA
GFAP	Mouse	1:400	2 h	Millipore, Darmstadt, Germany
β-APP	Rabbit	1:500	2 h	Invitrogen, Carlsbad, CA, USA
TBR1	Rabbit	1:500	1 h	Abcam, Cambridge, UK
Ctip2	Rat	1:500	1 h	Abcam

PDGFRα, platelet-derived growth factor receptor-α; GAP-43, growth-associated protein; APP, amyloid precursor protein; GFAP, glial fibrillary acidic protein; NG2, neuron-glial antigen 2; MBP, myelin basic protein; TBR1, T-box brain 1.
